# Effects of Adding a Structured Exercise Programme to Ergonomics Training in Women Dairy Farmers: A Randomised Controlled Trial

**DOI:** 10.3390/healthcare14203381

**Published:** 2026-10-10

**Authors:** Ömer Şevgin, Muharem Akar, Suzan Aydın

**Affiliations:** 1 Department of Physiotherapy and Rehabilitation, Faculty of Health Sciences, Üsküdar University, 34768 Istanbul, Türkiye; muharemakar34@gmail.com; 2 Department of Physiotherapy and Rehabilitation, Faculty of Health Sciences, İstanbul Gelişim University, 34310 Istanbul, Türkiye

**Keywords:** farmers, dairying, musculoskeletal diseases, occupational diseases, occupational health, ergonomics, exercise therapy, quality of life

## Abstract

**Background/Objectives:** Milking exposes women dairy farmers to sustained trunk flexion and repetitive upper-limb loading, producing a high burden of musculoskeletal disorders. Ergonomics education is the conventional preventive strategy, but added benefit from structured exercise is untested in this population. **Methods:** In this assessor-blinded randomised controlled trial, 50 women who had milked cows for ≥5 years were allocated to ergonomics training alone (control, *n* = 25) or ergonomics training plus a 12-week, home-based, equipment-free exercise programme (experimental, *n* = 25). The primary outcome was pain intensity (NRS), designated as primary only after data collection, so all analyses are exploratory; secondary outcomes were musculoskeletal symptoms (Nordic Questionnaire, seven-day recall at both assessments), upper-limb function (QuickDASH), depression (BDI) and quality of life (WHOQOL-BREF). Continuous outcomes were analysed by mixed-model ANOVA and ANCOVA. **Results:** All participants completed the trial, with self-reported exercise adherence of 100%. Time × group interactions favoured the combined intervention for pain (F_1,48_ = 55.19, *p* < 0.001; baseline-adjusted mean difference −1.25, 95% CI −1.58 to −0.92), upper-limb function, depressive symptom scores and three WHOQOL-BREF domains (all *p* < 0.05), but not social relationships (*p* = 0.896). Mean NRS reduction was −3.76 ± 0.66 versus −2.24 ± 0.78 (d = −2.10), and symptomatic body regions fell by −2.64 ± 0.70 versus −1.20 ± 1.53 (*p* < 0.001). **Conclusions:** Both groups improved, and adding a 12-week equipment-free exercise programme was associated with greater short-term, self-reported gains. These exploratory findings from a small single-region sample need confirmation in larger trials with objective adherence measures and longer follow-up.

## 1. Introduction

Agriculture remains one of the occupational sectors with the highest burden of work-related musculoskeletal disorders (WMSDs). Heavy physical workload, repetitive movement, awkward and sustained postures, manual load handling, whole-body vibration and limited access to occupational health services combine to produce prevalence estimates that consistently exceed those of most industrial settings [1,2,3]. A systematic review of farming populations in Southeast Asia reported twelve-month prevalence figures well above general-population norms across virtually every body region [2], and a systematic review and meta-analysis restricted to low- and middle-income countries reached the same conclusion, with low back pain the single most frequently affected site [3]. National surveillance data point in the same direction: among Korean farmers, musculoskeletal disorders are now the most commonly recorded work-related disease, and their recorded prevalence rose between 2020 and 2022 [4].

The pattern is consistent across farming systems and continents. Elevated symptom rates have been documented in rice farmers in Thailand and Bangladesh [5,6], tobacco farmers in Thailand and India [7,8], tea harvesters [9], pineapple growers and mixed smallholders in north-east India [10,11], traditional farming communities in Rajasthan [12], tractor operators [13], tomato farmworkers [14] and farmers and ranchers in the United States Central States region [15]. A recent cross-sectional study reported that female sex, body mass index, type of farming, daily lifting, weekly working hours and psychological stress were all independently associated with musculoskeletal disorder risk [16].

Dairy farming, and milking in particular, represents a distinctive exposure profile within agriculture. Milking involves prolonged trunk flexion and rotation, sustained shoulder abduction and elevation, repetitive and forceful gripping, and static loading of the cervical and lumbar spine, typically repeated twice daily throughout the year [17,18]. Epidemiological work in Swedish and German dairy herds found that 84–85% of milkers reported musculoskeletal symptoms in at least one body region, and that female sex was independently associated with increased symptom risk [18]. Recent work in dairy diversification has confirmed that load carrying, manual work and awkward postures remain the dominant drivers of pain, most often in the lower back and neck [19], and a 2025 review of repetitive motion tasks in dairy milkers emphasised the link between postural stress, tendon strain and injury rates while noting that ergonomic training and task modification can reduce that risk [20].

Women carry a disproportionate share of this burden. Female farmers and farm workers report substantially higher rates of neck and hand/wrist complaints than their male counterparts, a difference attributed to anthropometric mismatch with equipment designed around male body dimensions, the additional domestic and caregiving workload carried by rural women, and differences in task allocation [18,21]. In older female farmers, musculoskeletal pain is the most prevalent occupational health problem, and its severity is more strongly related to general wellbeing than to sleep problems or measured ergonomic exposure [21]. Reduced handgrip strength has likewise been associated with musculoskeletal disorders in farming populations, suggesting that diminished physical capacity is part of the causal picture rather than merely a consequence of it [22].

The consequences extend well beyond localised pain. Persistent musculoskeletal symptoms restrict upper-limb function and therefore the capacity to perform the very tasks on which household income depends. They are also consistently associated with reduced health-related quality of life [23] and with elevated psychological distress: common mental disorders are substantially more frequent among farmers with musculoskeletal complaints than among those without [24], and a scoping review concluded that the relationship between distress and WMSDs in agricultural workers is bidirectional [25]. Population-level data from Norway similarly show that farmers’ physical and mental health outcomes are closely intertwined [26]. In rural settings where health-seeking behaviour is low and continuing to work is not optional, this cycle can become entrenched.

Two broad preventive strategies exist, and they act on opposite sides of the same load–capacity equation. Ergonomics education reduces the mechanical demand imposed by the task by modifying working posture, workstation layout, task organisation and body mechanics. Engineering solutions follow the same logic and can be effective—redesigned harvest baskets [27], a modified climbing device for date palm workers [28] and postural interventions in tobacco cultivation [8] have all reduced measured ergonomic risk—but they require equipment investment that many smallholders cannot make. Structured exercise programmes act on the other side of the equation: they increase muscular strength and endurance, restore range of motion and improve postural control, thereby raising the individual’s tolerance of whatever occupational load remains. Because the two approaches are mechanistically complementary, there is a strong rationale for combining them.

Empirical support for this combination is accumulating but remains markedly thinner in agriculture than in other sectors. An exploratory systematic review of non-surgical interventions in farmers concluded that participatory ergonomic intervention, strength-based exercise, or a combination of both could reasonably form the basis of preventive programmes, while noting the scarcity of high-quality randomised trials in this population [29]; the same gap prompted a dedicated systematic review protocol on ergonomic intervention in agricultural workers [30] and a scoping review protocol focused on vulnerable farming communities [31]. Where trials do exist in agriculture, they are encouraging: a personalised self-care programme reduced WMSDs among Thai rubber farmers [32]. Evidence from other occupational groups is stronger. A 2025 systematic review and meta-analysis found that ergonomic interventions reduce work-related musculoskeletal pain overall, with the largest effects for multi-component programmes that include an active training element [33]. Workplace exercise programmes reduce musculoskeletal pain and improve functional capacity in randomised trials [34,35,36], and a 12-week combined ergonomic training and exercise programme in supermarket cashiers produced greater reductions in musculoskeletal pain and ergonomic risk than ergonomic training alone [37].

To our knowledge, no randomised controlled trial has evaluated a combined ergonomics-and-exercise intervention specifically in women engaged in dairy milking, despite this being one of the most heavily exposed and least studied occupational groups. Reviews of ergonomic risk assessment in farmers also note that intervention studies in this sector rarely extend beyond posture measurement to patient-reported outcomes such as function, mood and quality of life [38]. The aim of this trial was therefore to compare the effects of ergonomics training alone with ergonomics training combined with a 12-week structured exercise programme on pain intensity, musculoskeletal symptom prevalence, upper-limb function, depressive symptoms and quality of life in women dairy farmers actively engaged in milking. We hypothesised that the combined intervention would produce greater improvement than ergonomics training alone across all outcome domains.

## 2. Materials and Methods

### 2.1. Study Design

This was a prospective, two-arm, parallel-group, assessor-blinded randomised controlled trial with a 1:1 allocation ratio and outcome assessment at baseline (week 0) and after the intervention (week 12). The study was conducted in rural districts of Bitlis Province, Türkiye. The trial was registered prospectively at ClinicalTrials.gov (NCT07442409); the registration was first submitted on 25 February 2026, four days before the study start date of 1 March 2026 and before enrolment of the first participant. Recruitment began on 1 March 2026, and the final week-12 assessment was completed in the week of 15 July 2026. Reporting follows the CONSORT 2025 statement for parallel-group randomised trials [39].

### 2.2. Participants

Women actively engaged in dairy milking were recruited from rural communities in Bitlis and its affiliated districts. Recruitment proceeded through field visits by the research team to villages in the study area: the study was announced through village headmen (muhtars) and local primary health-care units, and women who volunteered were screened for eligibility on site. Participants milked cows by hand, performing on average approximately two hours of manual milking per day. Inclusion criteria were female sex; age 25–60 years; active engagement in milking for at least five years; sufficient physical capacity to participate in the planned education and exercise programme; and provision of written informed consent. The age range of 25–60 years was chosen to capture women of working age with an established milking history of at least five years, while limiting the confounding influence of age-related degenerative musculoskeletal change, which becomes increasingly prevalent beyond 60 years of age. Exclusion criteria were any diagnosed concomitant musculoskeletal or rheumatological disease affecting any body region—including, given the manual nature of milking, diagnosed conditions of the wrist and hand such as carpal tunnel syndrome or tendinopathy—identified at the screening interview; any concomitant neurological disease or neurological dysfunction; a history of surgery involving the musculoskeletal system; and any serious systemic disease that would preclude participation.

### 2.3. Randomisation and Blinding

After completion of the baseline assessment, participants were allocated in a 1:1 ratio to the experimental or control group using simple unrestricted computer-generated randomisation without blocking or stratification; therefore, no block size was applicable. The allocation sequence was generated before enrolment by an independent researcher who had no role in participant recruitment, baseline assessment, treatment delivery, outcome assessment, or statistical analysis. Group assignments were placed in sequentially numbered, opaque, sealed envelopes prepared according to the generated sequence. The envelopes were opened consecutively only after the participant had been enrolled and the baseline assessment had been completed. Following completion of the study, the original randomisation sequence and allocation log were independently cross-checked by a researcher who had not participated in recruitment, treatment delivery, outcome assessment, or statistical analysis. Because of the nature of the intervention, neither participants nor the therapist delivering the programme could be blinded. The outcome assessor was blinded to group allocation and remained blinded throughout the baseline and week-12 assessments; the trial was therefore single-blind (assessor-blinded).

### 2.4. Interventions

#### 2.4.1. Control Group: Ergonomics Training

Participants in the control group received a standardised ergonomics training programme addressing the ergonomic risk factors encountered during milking. Content covered appropriate body mechanics, correct working posture, seat and equipment positioning, load handling, organisation of work and rest cycles, and general principles of musculoskeletal protection. Specifically, the first session addressed the milking posture itself: sitting on a stable low stool with the trunk as close to neutral as possible rather than squatting or kneeling, positioning the stool and the container close to the animal to avoid sustained trunk flexion combined with rotation, alternating the leading hand, and keeping the wrist in a neutral position with the forearm supported where possible. The second session focused on load handling and work organisation: carrying milk containers with both hands and close to the body, avoiding lifting with a flexed and rotated spine, dividing heavy loads, changing position briefly between animals, and inserting short pauses with simple stretches into the milking routine. The third session reviewed workstation and environmental arrangements—stool height, container placement, flooring and lighting—and reinforced the earlier content, with individual correction of each participant’s habitual postures as observed and reported.

The training was delivered individually and face to face on three occasions—at the start of the intervention, at the end of week 4 and at the end of week 8—each session lasting approximately one hour, giving about three hours of one-to-one ergonomics education per participant. Sessions were structured around a slide presentation supported by photographs and video demonstrations contrasting correct and incorrect working postures during milking, and every participant was given a printed illustrated brochure summarising the principles covered, which she kept for reference. Repeating the session at weeks 4 and 8 allowed the recommendations to be reinforced and adapted to each participant’s own milking set-up. In addition to the three face-to-face sessions, all participants in the control group—like those in the experimental group—received a telephone call from the study team every two weeks throughout the 12-week period; these calls asked only about general status and study retention and did not include any exercise-specific content, so that overall contact frequency was matched between groups while contact content differed (Section 4.4). All ergonomics training and all exercise instruction were delivered by the same physiotherapist.

#### 2.4.2. Experimental Group: Ergonomics Training Plus Exercise

Participants in the experimental group received exactly the same ergonomics training as the control group—three individual, face-to-face sessions of approximately one hour at baseline and at the end of weeks 4 and 8—plus a structured exercise programme of stretching, strengthening and postural exercises targeting the neck, shoulder girdle, thoracic spine and lumbopelvic region. The programme followed the principles of progressive loading, muscle-group-specific exercise selection, standardised set and repetition schedules and standardised rest intervals. Exercises were taught face to face by the physiotherapist and continued as a home programme three days per week on non-consecutive days for 12 weeks. The two intervention components thus differed intentionally in application frequency: the ergonomic recommendations were to be applied continuously, during every daily milking session and related farm tasks, whereas the structured exercise programme was performed on three days per week.

The programme was delivered as two alternating session types performed on three non-consecutive days per week, so that each participant completed 36 sessions over the 12 weeks. Session A targeted the cervical spine, scapula and upper limb; session B targeted the thoracic spine, trunk and lower limb. Each session opened with the same five-minute warm-up (marching in place, shoulder rolls, arm circles and active cervical range-of-motion movements) and continued with a stretching block, performed throughout the programme as two sets of 30 s per stretch, targeting the muscle groups shortened by the milking posture. Session A then comprised a postural and scapular control block (chin tuck, scapular setting, wall angels, prone Y–T–W) followed by upper-limb and scapular strengthening (push-up progression, prone scapular row, isometric grip work). Session B comprised quadruped thoracic rotation followed by trunk and lower-limb strengthening (squat progression, hip hinge and bridge progression, dead bug and bird-dog, side plank progression and standing calf raise). Repetitions were increased progressively across the three four-week phases and a third set was added in the final phase, so that session duration rose from approximately 40 min in phase 1 to approximately 50 min in phase 3. All exercises were performed with body weight alone; no elastic bands, weights or other equipment were used at any stage, so the programme required nothing that a participant did not already have at home.

The programme was progressed in three four-week phases according to the principle of progressive overload. In phase 1 (weeks 1–4) participants performed regressed variants of each exercise for two sets of 10 repetitions; in phase 2 (weeks 5–8), intermediate variants for two sets of 15 repetitions; and in phase 3 (weeks 9–12), advanced variants for three sets of 8 repetitions with longer isometric holds. Stretches were held for 30 s and performed as two sets throughout all three phases. Rest intervals were standardised at 30 s between sets and 45 s between exercises. Progression to the next variant was permitted only when the participant could complete all prescribed sets with correct technique at a rating of perceived exertion of 5 or below on the Borg CR10 scale and without an increase in pain exceeding two points on the NRS persisting beyond 24 h. The full programme is presented in Table 1. The intervention is reported in accordance with the Consensus on Exercise Reporting Template (CERT) and the Template for Intervention Description and Replication (TIDieR).

**Table 1:** (**a**) Session A (cervical spine, scapula and upper limb) of the twelve-week structured exercise programme delivered to the experimental group, reported in accordance with the Consensus on Exercise Reporting Template (CERT) and the Template for Intervention Description and Replication (TIDieR). The two session types were alternated over three sessions per week. (**b**) Session B (thoracic spine, trunk and lower limb) of the twelve-week structured exercise programme delivered to the experimental group.

**(a)**				
**Component**	**Exercise**	**Phase 1 (Weeks 1–4)**	**Phase 2 (Weeks 5–8)**	**Phase 3 (Weeks 9–12)**
Warm-up (5 min)	Marching in place	2 min	2 min	3 min
	Shoulder rolls and arm circles	10 each direction	12 each direction	15 each direction
	Active cervical range of motion (flexion–extension, rotation, side-bend)	5 each direction	8 each direction	10 each direction
Stretching (2 × 30 s throughout)	Upper trapezius stretch	2 × 30 s each side	2 × 30 s each side	2 × 30 s each side
	Levator scapulae stretch	2 × 30 s each side	2 × 30 s each side	2 × 30 s each side
	Doorway pectoral stretch	2 × 30 s	2 × 30 s	2 × 30 s
	Wrist flexor and extensor stretch	2 × 30 s each	2 × 30 s each	2 × 30 s each
Postural/scapular control	Chin tuck	2 × 10, 5 s hold	2 × 15, 5 s hold	3 × 8, 8 s hold
	Scapular setting (retraction and depression)	2 × 10, 5 s hold	2 × 15, 5 s hold	3 × 8, 8 s hold
	Wall angels	2 × 10	2 × 15	3 × 8
	Prone Y–T–W	2 × 8 each position	2 × 12 each position	3 × 8 each position
Calisthenic strengthening—upper limb and scapula	Push-up progression	Wall push-up, 2 × 10	Incline/knee push-up, 2 × 15	Knee or full push-up, 3 × 8
	Prone scapular row	Isometric scapular squeeze, 2 × 10	Prone row, 2 × 15	Prone row with 3 s hold, 3 × 8
	Isometric grip work	2 × 10, 3 s hold	2 × 15, 5 s hold	3 × 8, 8 s hold
(**b**)				
**Component**	**Exercise**	**Phase 1 (Weeks 1–4)**	**Phase 2 (Weeks 5–8)**	**Phase 3 (Weeks 9–12)**
Warm-up (5 min)	Marching in place	2 min	2 min	3 min
	Shoulder rolls and arm circles	10 each direction	12 each direction	15 each direction
	Active cervical range of motion	5 each direction	8 each direction	10 each direction
Stretching (2 × 30 s throughout)	Latissimus dorsi stretch (overhead side reach)	2 × 30 s each side	2 × 30 s each side	2 × 30 s each side
	Thoracic extension over chair back	2 × 30 s	2 × 30 s	2 × 30 s
	Hamstring stretch (supine or seated)	2 × 30 s each side	2 × 30 s each side	2 × 30 s each side
	Half-kneeling hip flexor stretch	2 × 30 s each side	2 × 30 s each side	2 × 30 s each side
Mobility/motor control	Quadruped thoracic rotation	2 × 8 each side	2 × 12 each side	3 × 8 each side
Calisthenic strengthening—trunk and lower limb	Squat progression	Sit-to-stand from chair, 2 × 10	Bodyweight squat, 2 × 15	Squat with 3 s eccentric, 3 × 8
	Hip hinge and bridge progression	Glute bridge, 2 × 10	Hip hinge + bridge, 2 × 15	Single-leg bridge, 3 × 8
	Dead bug/bird-dog	Dead bug, 2 × 10 each side	Bird-dog, 2 × 15 each side	Bird-dog with 3 s hold, 3 × 8 each side
	Side plank progression	Knee side plank, 2 × 15 s each side	Knee side plank, 2 × 25 s each side	Full side plank, 3 × 30 s each side
	Standing calf raise	2 × 13	2 × 18	3 × 11

Frequency: three sessions per week on non-consecutive days for 12 weeks, alternating between session types A and B (36 sessions in total). Session duration: approximately 40 min in phase 1, rising to approximately 50 min in phase 3. Setting: home-based, after face-to-face instruction by the physiotherapist, with telephone contact every two weeks to ask about completion and review technique. Rest intervals: 30 s between sets and 45 s between exercises. Progression criterion: a participant advanced to the next phase variant only when she could complete all prescribed sets with correct technique at a rating of perceived exertion of 5 or below on the Borg CR10 scale and without an increase in pain of more than two points on the Numerical Rating Scale persisting beyond 24 h. Tailoring: exercises could be regressed to the preceding phase variant at any point if pain increased or technique deteriorated. All exercises were performed with body weight alone; no elastic bands, weights or other equipment were used at any point in the programme.

Adherence was monitored by telephone contact every two weeks throughout the 12-week period. During each call, the physiotherapist asked the participant to report whether the prescribed sessions of the preceding two weeks had been completed on non-consecutive days, checked for pain or difficulty with the current phase variant, and answered questions about technique. This monitoring relied entirely on participant self-report; no exercise diary, video recording, application log or in-person technique check was used to verify completion or quality of performance objectively. On this basis, all participants in the experimental group self-reported having completed all 36 prescribed sessions (three per week for 12 weeks), giving a self-reported adherence rate of 100%; this figure should be interpreted in light of the absence of objective verification. No participant in either group withdrew or was lost to follow-up.

### 2.5. Outcome Measures

All measures were administered by face-to-face interview at baseline and repeated at week 12 using an identical protocol. All participants were literate; interviews followed a standard question order and wording for every participant, and the interviewer provided additional plain-language explanation of individual items on request without altering their intended meaning, to ensure comprehension was consistent across participants with differing educational backgrounds. A demographic information form recorded age, height, body weight, educational level and marital status.

#### 2.5.1. Primary Outcome

Pain intensity was assessed using the Numerical Rating Scale (NRS), a unidimensional scale on which the participant rates perceived pain from 0 (no pain) to 10 (unbearable pain). Participants were asked to rate their general work-related musculoskeletal pain—considering the neck, shoulders and lower back together, the regions most consistently implicated in milking—rather than a single anatomically restricted site; where more than one region was symptomatic, participants rated their overall pain intensity rather than any one region in isolation. The assessment was made at the time of the study visit and referred to the participant’s usual or typical level of pain rather than pain at a single instant. No minimum NRS score was required for study entry; the inclusion criteria specified occupational exposure and physical capacity to participate rather than a symptom threshold, and observed baseline pain levels reflect the naturally high symptom burden of this occupational group rather than a selection criterion. Its validity as a ratio measure of pain intensity has been established [40]. We acknowledge that this validation concerns pain-intensity measurement in general rather than the aggregation of several body regions into a single rating. A single overall rating was nonetheless designated as the primary outcome because the exposure of interest—milking—loads the neck, shoulders and lower back simultaneously and typically produces overlapping, multi-regional pain, so that any single region-specific score would have captured only part of the construct of interest; region-specific information was collected separately with the Nordic Questionnaire (Section 2.5.2). The use of a single aggregated rating is acknowledged as a measurement limitation in Section 4.4. This designation was made after data collection: the trial registration in force during recruitment listed four co-primary outcomes (Nordic Questionnaire, QuickDASH, Beck Depression Inventory and WHOQOL-BREF) and did not include the NRS. The reclassification of the NRS as the primary outcome and of the four registered measures as secondary outcomes is therefore post hoc and is reported as a protocol deviation in Section 2.5.3. All outcomes are accordingly interpreted as exploratory. The scale was administered verbally as an 11-point numerical rating scale: participants were asked to state a whole number between 0 and 10, rather than to mark a position on a 10 cm line. The instrument is therefore a numerical rating scale rather than a visual analogue scale and is referred to as such throughout. The trial registration and earlier descriptions of this study referred to it as a visual analogue scale; that was imprecise terminology for the instrument actually used, and no change to the data is implied.

#### 2.5.2. Secondary Outcomes

Musculoskeletal symptoms were recorded with the Standardised Nordic Musculoskeletal Questionnaire, which documents trouble (ache, pain or discomfort) in nine body regions: neck, shoulders, upper back, elbows, wrists/hands, lower back, hips/thighs, knees and ankles/feet. The Turkish version has been validated [41]. The seven-day section of the questionnaire was administered at both assessments, so that baseline and week-12 prevalence refer to the same recall window and are directly comparable. Each region was coded as symptom present or absent at each time point, and a total symptom count (0–9 regions) was derived.

Upper-limb functional status was assessed with the Quick Disabilities of the Arm, Shoulder and Hand questionnaire (QuickDASH), an 11-item instrument scored from 0 to 100 in which higher scores indicate greater functional disability. The Turkish version has been shown to be valid and reliable [42].

Depressive symptoms were quantified with the Beck Depression Inventory (BDI), a self-report instrument in which higher total scores indicate more severe symptoms [43]; the Turkish version has been shown to be valid and reliable [44].

Quality of life was assessed with the World Health Organization Quality of Life Instrument—Short Form (WHOQOL-BREF), which yields four domain scores (physical health, psychological health, social relationships and environment) together with two separate items on overall quality of life and satisfaction with health (Q1 and Q2). Domain scores were computed from their constituent items and transformed to the 4–20 metric per the WHOQOL-BREF scoring manual, with higher scores indicating better quality of life. Because Q1 and Q2 are not combined into a domain score in the standard scoring algorithm, we additionally report a general two-item facet score, calculated as the mean of the raw Q1 and Q2 item scores and transformed to the same 4–20 metric as the four domains for comparability of presentation. This general two-item facet is descriptive rather than a validated WHOQOL-BREF domain; it is referred to as the “general two-item facet” throughout the text, tables and figures to distinguish it from the four validated domains and is interpreted accordingly. The psychometric properties of the Turkish version have been established [45].

#### 2.5.3. Deviations from the Registered Protocol

Four deviations from the registered protocol are reported. First, the Cornell Musculoskeletal Discomfort Questionnaire, listed among the planned instruments in the approved protocol, was not administered. This decision was made before data collection began, at the protocol-finalisation stage, and was not influenced by any subsequently observed data; the Nordic and Cornell questionnaires assess the same construct, the Nordic instrument was judged sufficient for the regional symptom mapping required by the study aims, and omitting the Cornell questionnaire also reduced respondent burden. Second, the trial registration in force during recruitment (first posted 2 March 2026) listed the Nordic Questionnaire, QuickDASH, Beck Depression Inventory and WHOQOL-BREF as four co-primary outcomes and did not list the Numerical Rating Scale; the designation of the NRS as the single primary outcome and of the remaining measures as secondary outcomes was made after data collection and is post hoc. The registry record was amended accordingly on 27 August 2026. Third, the registry entry specified an upper age limit of 50 years, whereas the approved protocol and the eligibility criteria applied in the field specified 25–60 years; the registry figure was a clerical error and was corrected in the same amendment. Eight participants (16%) were aged 51–60, and a sensitivity analysis excluding them is reported in Section 3.7. Fourth, the registry recorded an outcome time frame of 14 weeks, whereas outcomes were assessed at week 12 as described above. Because of the first two deviations in particular, all analyses in this report should be read as exploratory rather than confirmatory.

### 2.6. Sample Size

Sample size was calculated a priori with G*Power version 3.1.9.7 (Heinrich-Heine-Universität Düsseldorf, Düsseldorf, Germany), using the “*t* tests—Means: Difference between two independent means (two groups)” test family and statistical test. No directly comparable prior randomised controlled trial was available for this specific population and intervention (see Section 1), so the calculation assumed a large effect size (*d* = 0.80) following Cohen’s conventional benchmark for a large effect [46] rather than an effect size drawn from pilot or prior trial data; this assumption is revisited in the sensitivity analysis below. With this assumption, a two-tailed alpha of 0.05 and power (1 − β) of 0.85, a minimum total sample of 60 participants was indicated; no additional allowance for anticipated dropout was built into this figure. Although a margin of 5–10% is customary in clinical trials, the recruitable population here was fixed and already smaller than the calculated target, so inflating the target figure could not have yielded additional participants; close supervision in a small number of villages was also expected to minimise attrition. The absence of a prespecified dropout margin is nonetheless acknowledged as a deviation from usual practice (Section 4.4). The number of women who met the eligibility criteria, consented and were available for the full 12-week programme in the study region was 50. Recruitment was stopped at this point because the pool of eligible volunteers in the participating villages had been exhausted, not on the basis of any interim inspection of outcome data; extending recruitment to more distant districts was not feasible within the resources of the study. Of 54 women assessed for eligibility during the recruitment period, 4 did not meet the inclusion criteria and were excluded before randomisation, leaving 50 eligible participants who were randomised in equal numbers to the two groups (25 per group).

A sensitivity analysis was performed for the achieved sample. With 25 participants per group and a two-tailed alpha of 0.05, the smallest detectable between-group effect size is *d* = 0.87 at the 85% power assumed a priori, *d* = 0.94 at 90% power and *d* = 0.81 at 80% power. The realised power to detect the originally assumed effect of *d* = 0.80 was therefore 0.79 rather than the planned 0.85. The trial should accordingly be regarded as underpowered relative to its original design, and the point estimates for smaller between-group effects—particularly for secondary outcomes such as the WHOQOL-BREF social relationships domain, where no between-group difference was detected—should be interpreted with this reduced sensitivity in mind; a true but modest effect could have gone undetected. For this reason, between-group results are reported with 95% confidence intervals throughout, and interpretation rests on these interval estimates rather than on post hoc statements about achieved power.

### 2.7. Statistical Analysis

Analyses were performed on the 50 participants with complete baseline and week-12 data. Continuous variables are presented as mean ± standard deviation and categorical variables as frequency and percentage. Normality of continuous variables and of within-subject change scores was examined with the Shapiro–Wilk test.

Within-group pre–post comparisons used the paired-samples *t*-test where the distribution of change scores was normal and the Wilcoxon signed-rank test otherwise. Between-group comparisons of baseline values and change scores used the independent-samples *t*-test or the Mann–Whitney *U* test according to the same criterion. Categorical variables were compared with the chi-square test or, for 2 × 2 tables, Fisher’s exact test. Because the Nordic Questionnaire used the same seven-day recall window at both assessments (Section 2.5.2), regional symptom prevalence was compared within each group with McNemar’s exact test and between groups at week 12 with Fisher’s exact test. The total number of symptomatic regions was compared within groups with the Wilcoxon signed-rank test and between groups with the Mann–Whitney *U* test, the distribution of change scores being non-normal. No adjustment was made for the number of body regions tested, so the regional analyses are exploratory.

The combined effects of time (baseline versus week 12) and group (control versus experimental) were evaluated with 2 × 2 mixed-model (split-plot) analysis of variance, with the time × group interaction as the effect of primary interest. Because randomisation did not produce complete baseline equivalence on all variables, analyses of covariance were additionally performed on week-12 scores with the corresponding baseline score as covariate; the homogeneity-of-regression-slopes assumption was tested for each model and is reported alongside the ANCOVA results. For every continuous outcome, a single analysis was designated as the principal between-group comparison: the baseline-adjusted ANCOVA on week-12 scores, given the baseline imbalances noted below. Where the homogeneity-of-slopes assumption was violated for an outcome, the between-group comparison of change scores—with the mixed-model time × group interaction as a sensitivity analysis—took its place as the principal analysis for that outcome. All remaining analyses (within-group pre–post tests and the main effects of the mixed-model ANOVA) are reported as supporting, descriptive context rather than independent evidence of a treatment effect, to avoid giving excessive weight to multiple parallel tests. Analyses of secondary outcomes are exploratory and were not adjusted for multiplicity.

Effect sizes are reported as Cohen’s d for between-group comparisons of change scores, as *d_z_* or the matched-pairs rank-biserial correlation for within-group comparisons, and as partial eta squared for ANOVA and ANCOVA effects. Two-tailed *p* values below 0.05 were considered statistically significant. As a sensitivity analysis, all between-group comparisons were repeated after excluding the eight participants aged 51–60, that is, those outside the age range recorded in the trial registry at the time of recruitment. No adjustment was made for multiple comparisons across the secondary outcomes, which should therefore be interpreted as exploratory. Data were analysed using IBM SPSS Statistics version 26.0 (IBM Corp., Armonk, NY, USA).

## 3. Results

### 3.1. Participant Flow and Baseline Characteristics

**Figure 1:**
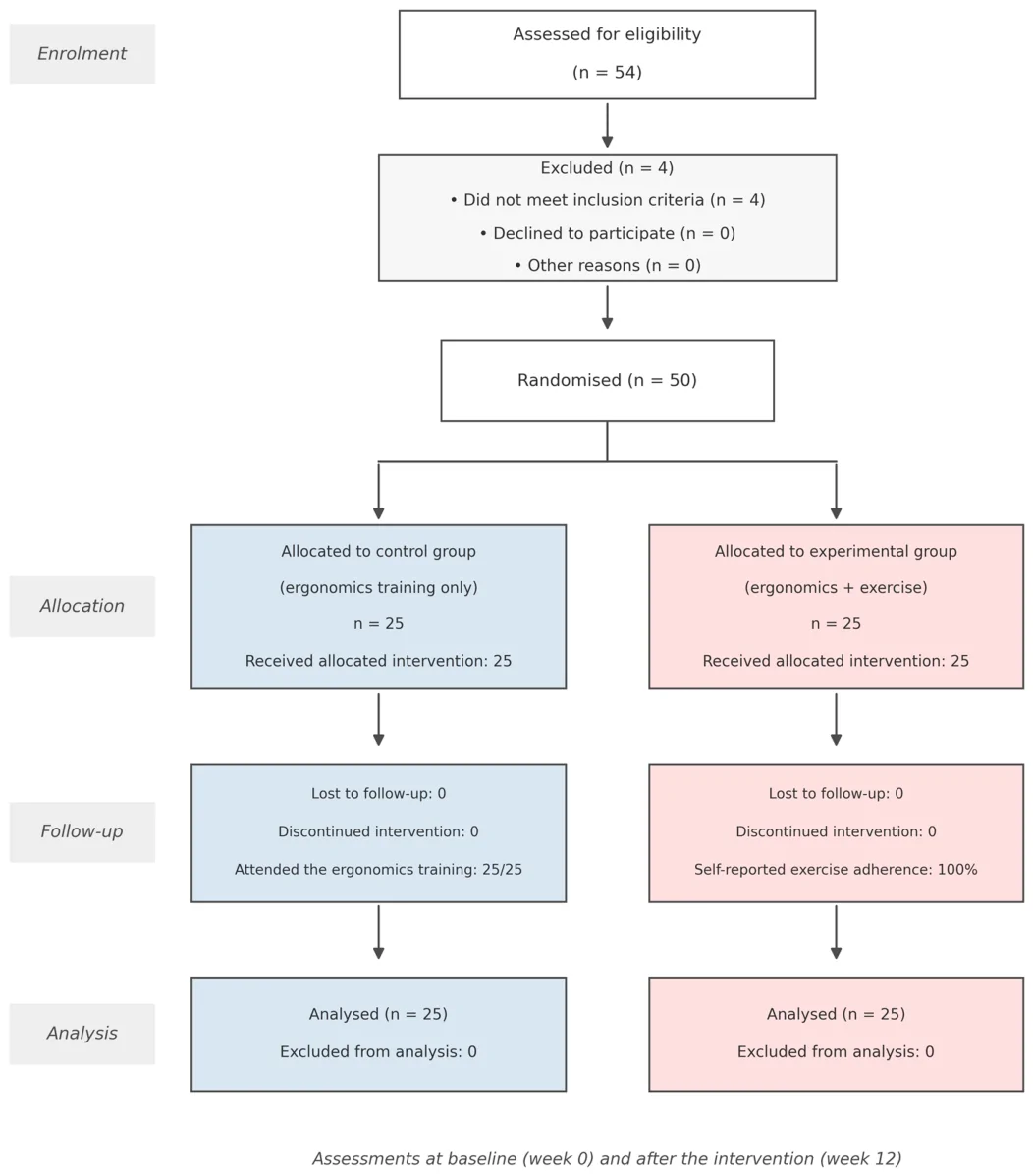
CONSORT flow diagram of participant progress through the trial. All randomised participants received their allocated intervention, all control participants attended the ergonomics training, all experimental participants self-reported completion of the prescribed exercise sessions, and no participant discontinued or was lost to follow-up. Blue boxes denote the control arm (ergonomics training only) and pink boxes the experimental arm (ergonomics training plus exercise); the shading distinguishes the two arms only and carries no other meaning.

Fifty women were randomised, 25 to the control group and 25 to the experimental group. No participant withdrew or was lost to follow-up; all participants in the experimental group self-reported completion of all 36 prescribed exercise sessions, and all 50 participants completed both the baseline and week-12 assessments (Figure 1). No intervention-related adverse events, exercise-related pain flares or injuries were reported by any participant in either group during the 12-week period. The mean age of the sample was 38.72 ± 10.82 years, and the mean body mass index was 28.38 ± 6.18 kg/m^2^. Baseline characteristics are presented in Table 2.

**Table 2:** Baseline demographic and anthropometric characteristics.

Variable	Control (*n* = 25)	Experimental (*n* = 25)	Test Statistic	*p*	SMD
Age (years)	37.88 ± 12.32	39.56 ± 9.26	*U* = 270.0	0.414	−0.15
Height (cm)	162.88 ± 4.85	158.56 ± 5.09	*t* = 3.072	0.004	0.87
Body weight (kg)	71.48 ± 12.81	74.36 ± 15.33	*U* = 273.5	0.454	−0.20
Body mass index (kg/m^2^)	26.95 ± 4.79	29.81 ± 7.12	*U* = 216.5	0.064	−0.47
Education †, *n* (%)			χ^2^ = 0.567	0.753	—
No formal/primary/secondary	13 (52)	11 (44)			
High school	4 (16)	6 (24)			
University/postgraduate	8 (32)	8 (32)			
Marital status, *n* (%)			Fisher exact	1.000	—
Married	18 (72)	18 (72)			
Single/other	7 (28)	7 (28)			

Values are mean ± standard deviation unless otherwise stated. Continuous variables were compared using the independent-samples *t*-test or the Mann–Whitney *U* test according to the Shapiro–Wilk test; categorical variables using the chi-square test or Fisher’s exact test. SMD, standardised mean difference (Cohen’s *d*, control minus experimental) for continuous variables. † The five original education categories (no formal education, primary/secondary school, high school, university, postgraduate) were collapsed into three groups for this comparison because several cells in the five-category table had expected counts below 5; all cells in the collapsed table have an expected count of at least 5. Despite a non-significant *p* value, body mass index showed a small-to-moderate standardised difference (*d* = −0.47) that a significance test alone would not flag; this is noted in the Discussion as a residual baseline consideration.

The groups did not differ significantly with respect to age, body weight, educational level or marital status. A statistically significant difference was found for height, with the control group taller than the experimental group (162.88 ± 4.85 cm versus 158.56 ± 5.09 cm, *p* = 0.004, SMD = 0.87); this is most plausibly a chance imbalance arising from simple randomisation in a modest sample, and height was not related to any outcome of interest. Body mass index did not differ significantly by conventional testing (*p* = 0.064), but the standardised mean difference (*d* = −0.47) indicates a small-to-moderate imbalance that the *p* value alone would understate; this is noted as a residual consideration when interpreting outcomes with a plausible weight dependence. At baseline, the groups also differed on two outcome variables: pain intensity was higher in the experimental group (4.80 ± 1.08 versus 4.16 ± 1.14, *p* = 0.039) and the WHOQOL-BREF physical domain score was higher in the experimental group (13.62 ± 1.35 versus 12.52 ± 2.03, *p* = 0.005). All other baseline outcome values were comparable (Table 3). Because of these imbalances, the ANCOVA results are reported alongside the mixed-model ANOVA results as the basis for between-group inference.

### 3.2. Primary Outcome: Pain Intensity

**Figure 2:**
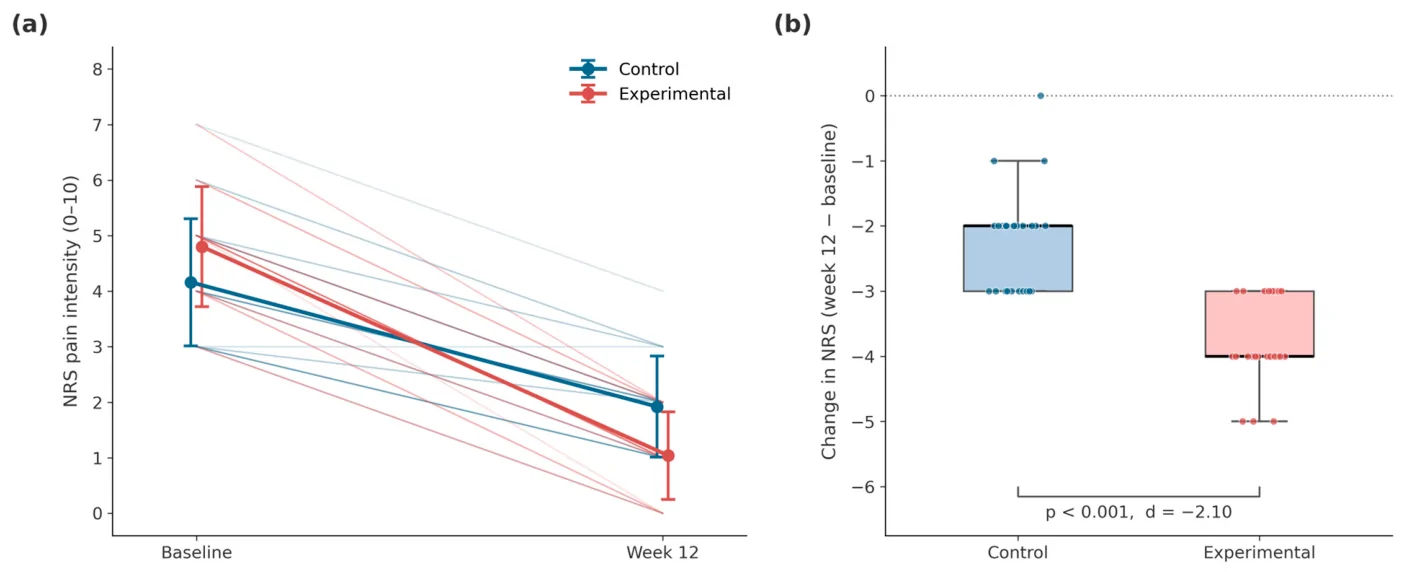
Primary outcome, pain intensity. (**a**) Individual trajectories (faint lines) with group means ± standard deviation at baseline and week 12. (**b**) Distribution of individual change scores by group; boxes show the median and interquartile range and points show individual participants. NRS, Numerical Rating Scale.

Pain intensity decreased significantly in both groups. In the control group, the NRS score fell from 4.16 ± 1.14 to 1.92 ± 0.91 (mean change −2.24 ± 0.78, *p* < 0.001), and in the experimental group, from 4.80 ± 1.08 to 1.04 ± 0.79 (mean change −3.76 ± 0.66, *p* < 0.001) (Figure 2).

The mixed-model ANOVA showed a large main effect of time (F_1,48_ = 859.87, *p* < 0.001, η_p_^2^ = 0.947), no main effect of group (F_1,48_ = 0.21, *p* = 0.647) and a large time × group interaction (F_1,48_ = 55.19, *p* < 0.001, η_p_^2^ = 0.535), indicating that the reduction in pain was significantly greater in the experimental group (Table 4). The between-group difference in change scores was −1.52 points (*p* < 0.001, Cohen’s *d* = −2.10). After adjustment for baseline pain, the experimental group scored 1.25 points lower at week 12 (95% CI −1.58 to −0.92, *p* < 0.001, η_p_^2^ = 0.551); the homogeneity-of-slopes assumption was satisfied (*p* = 0.970) (Table 5). Because the groups differed at baseline in pain (Table 3), the baseline-adjusted ANCOVA is the principal between-group comparison for this outcome, in line with the analysis strategy set out in Section 2.7; the unadjusted change-score difference and its effect size are reported as supporting context rather than as the primary estimate of effect.

### 3.3. Musculoskeletal Symptoms

**Figure 3:**
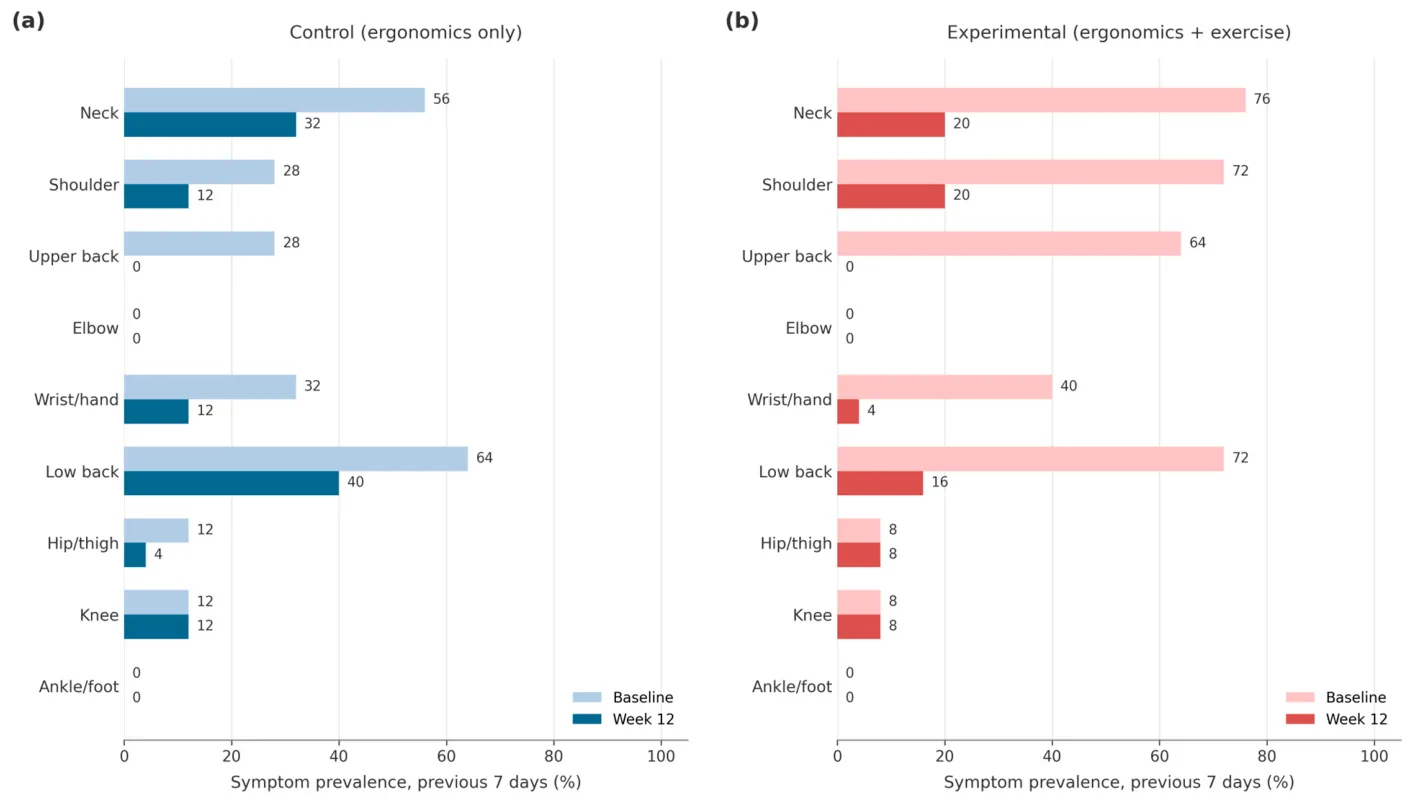
Prevalence of musculoskeletal symptoms in the previous seven days, by body region, at baseline and at week 12. (**a**) Control group (ergonomics training only). (**b**) Experimental group (ergonomics training plus exercise). Values beside the bars are percentages; zero values are labelled explicitly. In each panel, the light bars denote baseline and the dark bars week 12.

Symptom prevalence by body region is presented in Figure 3. At baseline, the most frequently affected regions were the lower back (64% of the control group and 72% of the experimental group), the neck (56% and 76%), the wrists/hands (32% and 40%), the shoulders (28% and 72%) and the upper back (28% and 64%), a distribution consistent with the exposure profile of milking work. No participant in either group reported elbow or ankle/foot symptoms at either assessment.

Symptom prevalence fell at week 12 in both groups. In the experimental group, the decline was statistically significant for the neck (76% to 20%, *p* < 0.001), the shoulders (72% to 20%, *p* < 0.001), the upper back (64% to 0%, *p* < 0.001), the lower back (72% to 16%, *p* < 0.001) and the wrists/hands (40% to 4%, *p* = 0.004). In the control group only the upper back reached significance (28% to 0%, *p* = 0.016); the reductions in the neck (56% to 32%, *p* = 0.070), lower back (64% to 40%, *p* = 0.146), wrists/hands (32% to 12%, *p* = 0.180) and shoulders (28% to 12%, *p* = 0.289) did not. Prevalence was essentially unchanged at the hip/thigh and knee in both groups, where baseline prevalence was already low. No participant in either group reported a new symptom in any region at week 12. Week-12 prevalence did not differ significantly between groups in any single region (Fisher’s exact test, all *p* ≥ 0.114), which is unsurprising given the number of participants with symptoms in each cell.

The total number of symptomatic body regions provides a compact summary. This fell from 2.32 ± 1.18 to 1.12 ± 0.67 regions in the control group (*p* = 0.003) and from 3.40 ± 0.65 to 0.76 ± 0.83 regions in the experimental group (*p* < 0.001). The reduction was significantly greater in the experimental group (−2.64 ± 0.70 versus −1.20 ± 1.53 regions; Mann–Whitney *U* test, *p* < 0.001; Cohen’s *d* = −1.21). The two groups also differed at baseline on this measure (*p* < 0.001), with a higher symptom burden in the experimental group, which should be borne in mind when interpreting the between-group difference in change (Section 4.4).

### 3.4. Upper-Limb Function

QuickDASH scores improved in both groups, from 25.36 ± 17.07 to 12.91 ± 11.05 in the control group (mean change −12.45 ± 16.29, *p* = 0.001, *d_z_* = −0.76) and from 33.73 ± 13.49 to 7.36 ± 11.33 in the experimental group (mean change −26.36 ± 14.69, *p* < 0.001, *d_z_* = −1.80). Baseline scores did not differ (*p* = 0.079). The improvement was significantly greater in the experimental group (between-group difference in change −13.91 points, *p* = 0.003, *d* = −0.90), and the time × group interaction was significant (F_1,48_ = 10.06, *p* = 0.003, η_p_^2^ = 0.173). After adjustment for baseline function, the experimental group scored 7.69 points lower at week 12 (95% CI −13.94 to −1.44, *p* = 0.017), with the homogeneity-of-slopes assumption satisfied (*p* = 0.978). A systematic review and meta-analysis proposed a minimal clinically important difference (MCID) of 12 to 15 QuickDASH points for people with musculoskeletal disorders [47]. Judged against this threshold, the relevant comparison for a randomised trial is the between-group difference rather than either group’s within-group change: the unadjusted between-group difference in change (−13.91 points) falls within the published MCID range, whereas the baseline-adjusted difference (−7.69 points) lies below it, so the additional benefit of exercise for upper-limb function should be interpreted as suggestive rather than clearly clinically important.

### 3.5. Depressive Symptoms

BDI scores were in the minimal range at baseline in both groups and decreased over the intervention period, from 6.68 ± 4.73 to 4.92 ± 3.89 in the control group (*p* < 0.001) and from 5.76 ± 3.24 to 2.24 ± 1.45 in the experimental group (*p* < 0.001, *d_z_* = −1.84). Baseline scores were comparable (*p* = 0.922). The reduction was significantly greater in the experimental group (between-group difference in change −1.76 points, *p* < 0.001, *d* = −1.13; interaction F_1,48_ = 15.91, *p* < 0.001, η_p_^2^ = 0.249). The homogeneity-of-slopes assumption was violated for the BDI ANCOVA (*p* < 0.001), so the mixed-model ANOVA and change-score analysis are the appropriate basis for inference for this outcome.

### 3.6. Quality of Life

**Figure 4:**
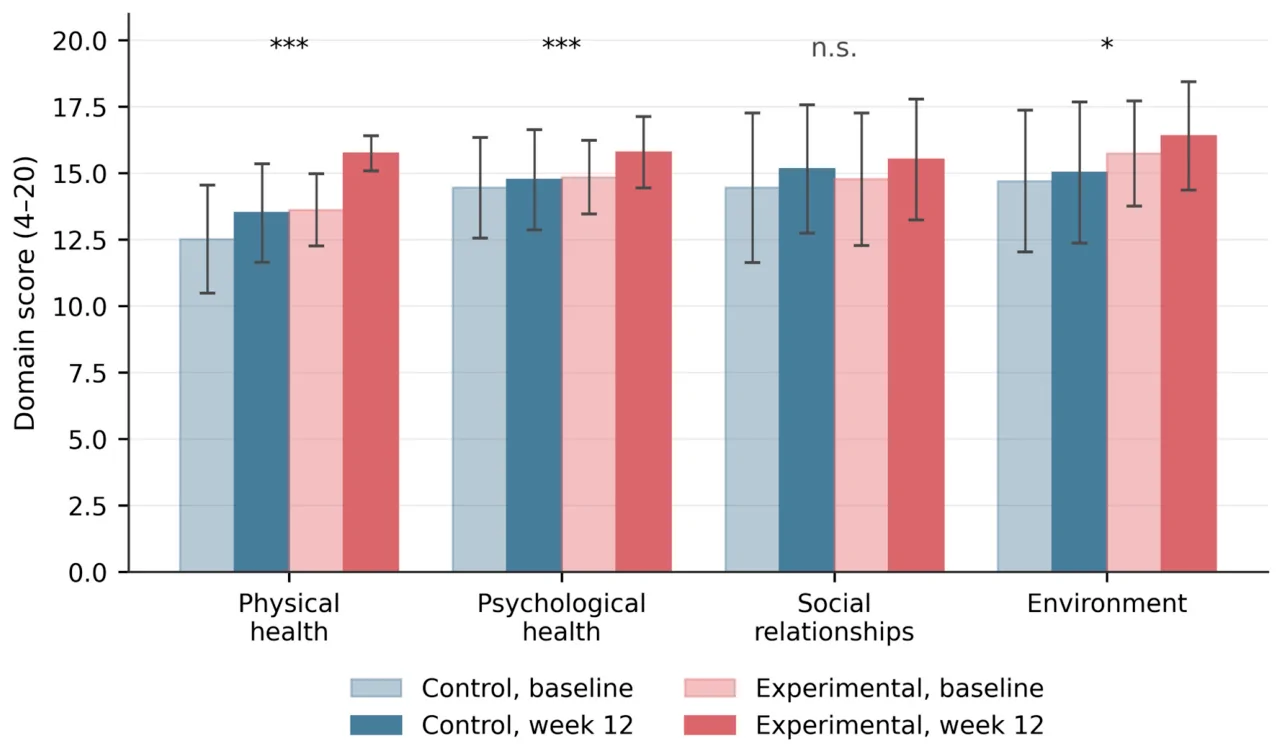
WHOQOL-BREF domain scores at baseline and week 12 by group. Bars show mean ± standard deviation on the 4–20 metric; higher scores indicate better quality of life. Symbols above each domain denote the time × group interaction: * *p* < 0.05; *** *p* < 0.001; n.s., not significant.

WHOQOL-BREF results are presented in Table 3 and Figure 4. Both groups improved significantly on the general two-item facet and on all four domains. Significant time × group interactions favouring the experimental group were found for the general two-item facet (*p* < 0.001), the physical health domain (*p* < 0.001), the psychological health domain (*p* < 0.001) and the environment domain (*p* = 0.017).

**Figure 5:**
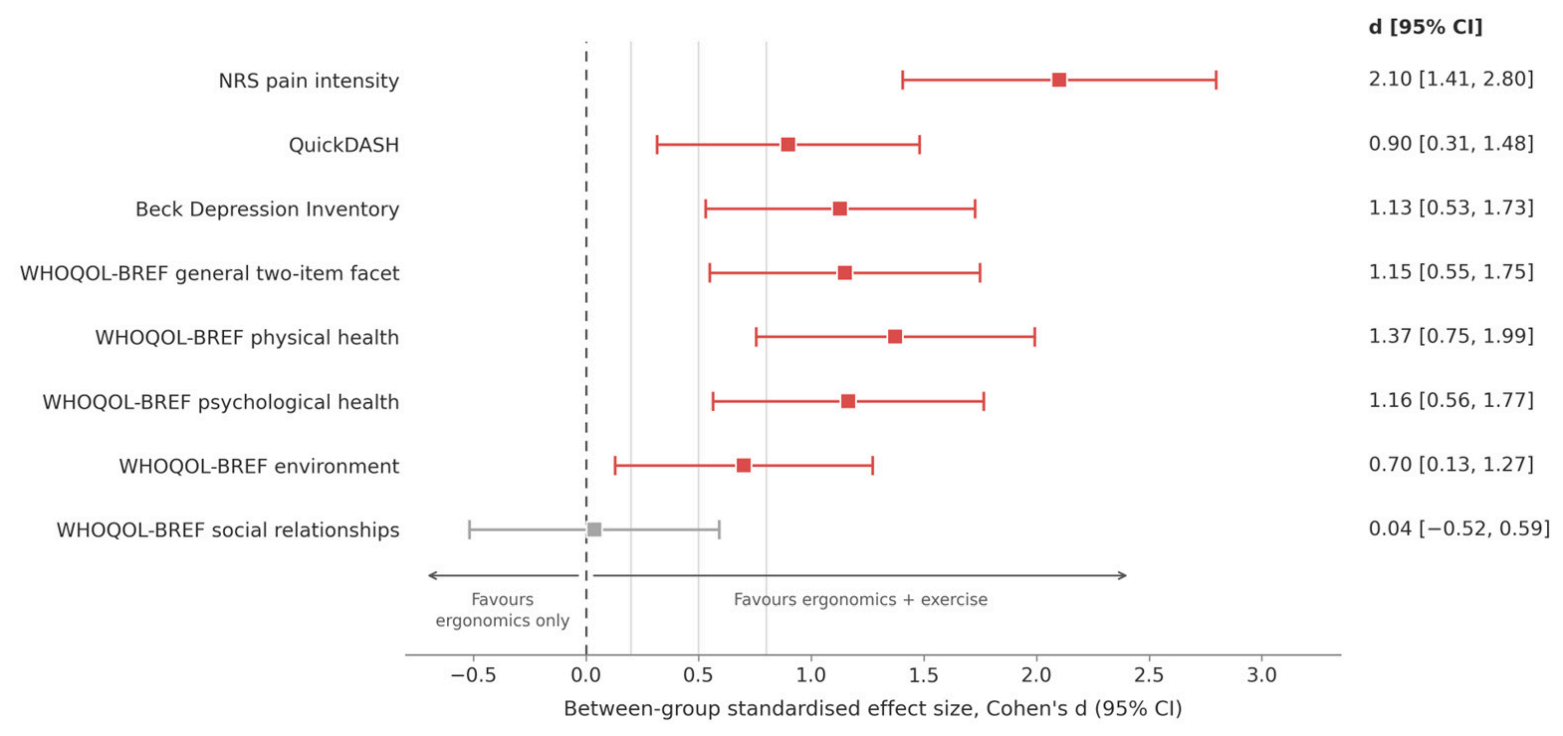
Between-group standardised effect sizes (Cohen’s *d* with 95% confidence intervals, CI) for the change from baseline to week 12. Effects are oriented so that positive values favour the combined ergonomics-and-exercise intervention. Squares are point estimates and horizontal bars the 95% CIs; red markers and bars denote intervals excluding zero and grey markers and bars intervals including zero. The vertical dashed line at 0 indicates no between-group difference, and the light grey vertical lines at 0.2, 0.5 and 0.8 mark Cohen’s conventional thresholds for small, medium and large effects. Nordic Questionnaire outcomes are omitted from the figure because they are binary regional variables rather than continuous scores; the corresponding between-group comparison for the total number of symptomatic regions is given in Section 3.3.

The social relationships domain was the single exception. Both groups improved (control: 14.45 ± 2.82 to 15.16 ± 2.42, *p* = 0.001; experimental: 14.77 ± 2.49 to 15.52 ± 2.27, *p* = 0.022), but the magnitude of improvement was virtually identical and neither the change-score comparison (*p* = 0.862, *d* = 0.04) nor the interaction term (F_1,48_ = 0.02, *p* = 0.896) approached significance. Among the WHOQOL-BREF outcomes, the largest between-group effect was seen in the physical health domain (interaction η_p_^2^ = 0.329; baseline-adjusted difference 1.47 points, 95% CI 1.07 to 1.87; as noted in Table 5, the homogeneity-of-slopes assumption was violated for this model, so the adjusted estimate should be interpreted with caution). The pattern of between-group standardised effect sizes across all outcomes is summarised in Figure 5.

### 3.7. Sensitivity Analysis for the Registered Age Range

All between-group comparisons were repeated after excluding the eight participants aged 51–60 (six control, two experimental), leaving 42 participants (19 control, 23 experimental). The pattern of results was unchanged. The time × group interaction remained significant for pain (F_1,40_ = 53.95, *p* < 0.001), upper-limb function (F_1,40_ = 12.58, *p* = 0.001), depressive symptoms (F_1,40_ = 16.05, *p* < 0.001) and the general (F_1,40_ = 11.18, *p* = 0.002), physical (F_1,40_ = 19.11, *p* < 0.001) and psychological (F_1,40_ = 12.51, *p* = 0.001) WHOQOL-BREF domains, and remained non-significant for social relationships (*p* = 0.598). The reduction in the number of symptomatic body regions remained significantly greater in the experimental group (*p* < 0.001). The single exception was the environment domain, for which the interaction was no longer significant in the reduced sample (F_1,40_ = 3.06, *p* = 0.088; full sample *p* = 0.017); this outcome should therefore be regarded as the least robust of the between-group differences reported here.

## 4. Discussion

This randomised controlled trial compared ergonomics training alone with ergonomics training combined with a 12-week structured exercise programme in women dairy farmers engaged in milking. Both interventions were followed by significant improvement across virtually every inferentially tested outcome, and the combined programme was superior on most of them. The time × group interaction was significant for pain intensity, upper-limb function, depressive symptoms and three of the four WHOQOL-BREF domains, with partial eta squared values between 0.11 and 0.54, and reported musculoskeletal symptom prevalence declined in both groups, more markedly in the combined group. The primary hypothesis was therefore supported for all inferentially tested outcomes except social relationships.

### 4.1. Pain and Musculoskeletal Symptoms

The baseline symptom profile mirrors what has been reported internationally for dairy work. Lower back, neck and shoulder complaints predominated, with seven-day prevalence in these regions ranging from 28% to 76%. Because most surveys of farming populations report twelve-month rather than seven-day prevalence, these figures are not directly comparable with the 84–85% twelve-month prevalence reported among Swedish and German milkers [18]; seven-day prevalence is expected to be substantially lower in the same population. The regional pattern does correspond: the predominance of the lower back and neck documented in dairy diversification work [19] and the elevated rates reported in women in agriculture [18,21] are both reproduced here. This convergence in pattern, if not in absolute level, supports the external validity of the sample.

The additional benefit of exercise over ergonomics alone is best understood in load–capacity terms. Ergonomics training reduces the mechanical demand imposed by the task; exercise raises the tissue’s tolerance of whatever demand remains. Where the fundamental task cannot be eliminated and equipment modification is constrained by cost, as in milking, the ceiling on what postural correction alone can achieve is low, and increasing capacity addresses the residual exposure directly. Our between-group effect on pain (*d* = −2.10) is directionally consistent with, though larger than, that reported in a comparable 12-week combined trial in supermarket cashiers [37], with an exploratory systematic review identifying participatory ergonomics, strength-based exercise or their combination as the most defensible basis for prevention in farmers [29], and with meta-analytic evidence favouring multi-component over single-component ergonomic interventions [33]. The one agricultural trial of comparable design, a personalised self-care programme for Thai rubber farmers, also reported meaningful symptom reduction [32], and the same pattern appears outside agriculture in back school programmes that pair education with active exercise [48,49]. The present trial extends that line of evidence to an agricultural occupational group.

The clinical, as distinct from statistical, relevance of the primary result also deserves comment. The baseline-adjusted between-group difference in pain of −1.25 points (95% CI −1.58 to −0.92) can be judged against the minimal clinically important change of approximately 1 point (or 15%) reported for chronic musculoskeletal pain intensity measured on an 11-point numerical rating scale [50], the same response format used here. The point estimate exceeds this threshold, and only the upper limit of the confidence interval (−0.92) falls marginally below it, so the additional analgesic benefit of exercise is likely to be clinically meaningful as well as statistically significant, although no population-specific threshold exists for women dairy farmers and this interpretation warrants corresponding caution. By contrast, as noted in Section 3.4, the baseline-adjusted between-group difference for upper-limb function lies below the published QuickDASH threshold, and the clinical importance of that effect remains suggestive rather than established. It is important not to overlook the magnitude of improvement in the control group. Mean pain there fell by 2.24 points and the number of symptomatic regions from 2.32 to 1.12. In a population with essentially no prior access to occupational health services, a low-cost educational intervention that achieves this is of considerable public health value in its own right, and it is the more scalable of the two options. This matters particularly in agriculture, where the engineering solutions that have proved effective elsewhere—redesigned harvest baskets, modified climbing devices, and mechanised aids [8,27,28]—require capital that smallholders in regions such as ours do not have. At the same time, the design does not permit the within-group improvement observed in the control group to be attributed to the ergonomics training itself: without an untreated comparison arm, regression to the mean, natural fluctuation of symptoms, seasonal variation in workload and non-specific effects of attention and study participation may all have contributed. The strongest causal inference this trial supports concerns the additional effect of exercise over ergonomics training alone, and the public-health interpretation of the control group’s improvement should be tempered accordingly.

### 4.2. Upper-Limb Function and Its Clinical Interpretation

The QuickDASH improvement in the experimental group was substantially larger than in the control group. This is mechanistically plausible: the exercise programme specifically targeted the neck, shoulder girdle and thoracic spine, the segments most directly implicated in the repetitive gripping and sustained shoulder elevation that characterise manual milking; upper-limb ergonomic risk in comparable agricultural tasks concentrates in exactly these segments [14]. Comparable functional gains have been reported following workplace exercise interventions in other occupational groups, including randomised trials of comprehensive workplace exercise in office workers [34] and of corrective exercise for upper crossed syndrome [36], and a systematic review of workplace exercise for musculoskeletal disorders reached the same conclusion [35]. Reduced handgrip strength has been linked to musculoskeletal disorders in farming populations [22], offering a plausible mechanism for the grip and forearm components of our programme.

Two features of this outcome deserve comment. First, the standard deviation of change in the control group (16.29 points) exceeded that in the experimental group (14.69 points) despite a smaller mean change, a pattern consistent with more variable responses to ergonomics advice alone; this difference in variability was not formally tested, however, and should be regarded as a descriptive observation rather than a confirmed effect. Second, the between-group effect size for upper-limb function (*d* = −0.90) was, apart from the WHOQOL-BREF environment domain, the smallest of the significant effects observed, and the corresponding partial eta squared for the interaction (0.173) was lower than that for pain (0.535). One possible explanation is that upper-limb disability responds over a longer timescale than pain intensity does, but this remains a hypothesis that our 12-week design cannot test directly.

### 4.3. Depressive Symptoms and Quality of Life

Improvements in BDI scores and in the physical, psychological and environmental WHOQOL-BREF domains favoured the combined intervention. This pattern is consistent with the bidirectional relationship between chronic musculoskeletal pain and psychological wellbeing described in agricultural populations [24,25]: reducing pain and restoring functional capacity plausibly improves mood and perceived quality of life. The finding also parallels cross-sectional evidence that work-related musculoskeletal disorders are inversely associated with quality of life in agricultural workers [23], and that musculoskeletal pain in older female farmers tracks general wellbeing more closely than measured ergonomic exposure [21]. Baseline BDI scores were in the minimal range in both groups, so the absolute change was small, and both groups remained within the minimal range at week 12. These findings should not be read as evidence of a clinically meaningful antidepressant effect; they describe a modest shift in self-reported mood within a non-clinical range, and a trial in participants with clinically relevant depressive symptoms would be required to address that question.

The absence of any between-group difference in the social relationships domain is coherent rather than anomalous. Social relationships in a rural farming community are shaped by family structure, community networks and economic circumstances, none of which either intervention was designed to alter, and the parallel within-group improvement in both groups more likely reflects the social contact inherent in participating in a supervised programme. This single null result is at most weak, indirect reassurance against a generalised response bias across the other self-reported outcomes, since the domain was not designated a priori as a specificity check.

### 4.4. Strengths and Limitations

The principal strengths of this study are its randomised, assessor-blinded controlled design with prospective trial registration; complete follow-up, with no withdrawals or losses in either group and self-reported completion of all prescribed exercise sessions, so that the analysis plausibly reflects the intervention as delivered rather than a diluted dose; the use of validated Turkish-language instruments throughout; a comprehensive outcome set spanning pain, symptoms, function, mood and quality of life; an intervention period of adequate duration; and its focus on a rural occupational group rarely represented in rehabilitation research.

Several limitations must be acknowledged. First, although the outcome assessor was blinded, participants and the treating physiotherapist necessarily were not, and all outcomes were self-reported; participant expectation may therefore have contributed to the magnitude of the observed effects. This concern is reinforced by the effect-size pattern for several outcomes (NRS pain and the WHOQOL-BREF physical and psychological domains and the general two-item facet, as well as the total number of symptomatic regions), for which the rank-biserial correlation was ±1.00, meaning that, once tied (unchanged) scores are excluded, every participant in the relevant group changed in the same direction with no exceptions; we re-verified this against the raw pre–post data and confirmed it is not a coding artefact. Such uniformity is plausible for a closely supervised, well-received programme in a small sample, but it is also the pattern one would expect from demand characteristics or social-desirability reporting, and it cannot be attributed to one explanation over the other with the unblinded, self-reported design used here. Both groups received telephone contact from the study team every two weeks to ask about general status, so the two arms were matched on contact frequency; the content of these calls nonetheless differed, since only the experimental group was asked about exercise completion and technique, and this residual difference in contact content cannot be excluded as a contributor to the observed between-group effects. Second, the analysed sample of 50 fell short of the 60 participants indicated by the a priori power calculation, reducing realised power for the assumed effect size from 0.85 to 0.79; this is unlikely to have affected the primary outcome but does mean that a genuine but modest between-group difference in the social relationships domain could have gone undetected. Third, despite randomisation, the groups differed at baseline in height, pain intensity, the WHOQOL-BREF physical domain and the number of symptomatic body regions, the last of these being a substantial imbalance (3.40 versus 2.32 regions, *p* < 0.001) in favour of a greater symptom burden in the experimental group; baseline-adjusted analyses were therefore performed for the continuous outcomes, although the homogeneity-of-slopes assumption was violated for three of them. Body mass index additionally showed a small-to-moderate but statistically non-significant imbalance (SMD = −0.47), which remains a residual consideration when interpreting outcomes with a plausible weight dependence. Because the Nordic outcome is binary at the regional level, no equivalent covariate adjustment was possible for it, and the larger reduction in symptomatic regions in the experimental group may partly reflect its higher baseline burden and the greater room for improvement that this allows. Fourth, adherence was established by participant self-report during fortnightly telephone calls rather than by an objective method such as an exercise diary, video recording or application log, so the reported adherence rate of 100% may overstate the dose actually performed; complete adherence with no attrition is in any case unusual in home-based exercise research and may reflect the close-knit rural setting and the small number of participating villages. The effects reported here should therefore be regarded as those obtainable under near-ideal delivery conditions. Fifth, no objective measure of ergonomic risk (such as REBA or RULA) or of physical capacity (such as grip strength or range of motion) was obtained; observational tools of this kind are the standard in agricultural ergonomics research [38], and their absence limits comparison with that literature. Sixth, the Cornell Musculoskeletal Discomfort Questionnaire specified in the approved protocol was not administered; although the Nordic questionnaire captures the same construct, the Cornell instrument would additionally have quantified symptom frequency and severity rather than mere presence. Seventh, the Nordic questionnaire records only the presence or absence of symptoms in the previous seven days and captures neither their frequency nor their severity, so a participant whose symptoms became less frequent or less intense without disappearing altogether is counted as unchanged; the regional analyses were also not adjusted for the number of body regions tested and should be regarded as exploratory. Eighth, participants were recruited from a limited number of rural communities and were individually rather than cluster randomised; participants from the same village or household could have discussed the ergonomics advice or exercises with one another, and any such contamination would tend to bias the between-group difference toward the null rather than inflate it. Ninth, beyond age, height, weight, body mass index, educational level and marital status, we did not collect a detailed occupational and clinical profile of participants—daily and weekly milking duration and frequency, herd size, working posture and seating arrangement, dominant hand, comorbid musculoskeletal conditions, analgesic or other medication use, seasonal variation in workload, or participation in other exercise—other than the fact that participants milked by hand for approximately two hours per day on average; such variables could plausibly modify both baseline symptom burden and responsiveness to intervention and should be captured in future trials in this population. Tenth, no adjustment was made for multiple testing across the secondary outcomes. Eleventh, the primary outcome was a single NRS rating of overall work-related musculoskeletal pain considering the neck, shoulders and lower back together; although this reflected the multi-regional exposure profile of milking and was complemented by region-specific Nordic data, such an aggregated rating has not been specifically validated for multi-region use and may mask region-specific differences in treatment response. Finally, there was no follow-up beyond the end of the intervention, so nothing can be said about whether the observed benefits are maintained, and the results were obtained in one province and may not generalise to other agricultural regions or farming systems.

### 4.5. Implications and Future Research

These findings suggest that occupational health provision for women in dairy farming should not stop at ergonomic advice. A home-based programme of three weekly sessions, performed entirely with body weight and requiring no equipment of any kind, produced additional benefit beyond ergonomics training alone for most outcomes, although, as noted above, the between-group difference for upper-limb function did not reach the published minimal clinically important difference. This last point is not incidental. The engineering interventions that have reduced ergonomic risk elsewhere in agriculture depend on capital that smallholders in regions such as this one do not have [8,27,28], and even elastic bands or hand weights represent a recurring cost and a supply problem in remote villages. A programme with no material requirements at all removes this barrier to dissemination through rural primary care or agricultural extension services, though a full costing would still need to account for the physiotherapist’s time and training; whether participants continue the exercises unsupervised beyond 12 weeks was not assessed in this trial and would require longer-term follow-up. This matters because the evidence base for intervention in agriculture remains thin relative to the size of the problem: recent reviews and review protocols continue to identify the shortage of randomised trials in farming populations as the principal gap [29,30,31]. Future trials should incorporate objective measures of ergonomic exposure [38] alongside objective measures of physical capacity such as handgrip strength [22]; objectively verified adherence rather than self-report; and follow-up of at least six to twelve months to establish whether the gains persist once supervision ends. Trials in male dairy farmers and in mixed-sex samples would clarify whether the effects observed here are specific to women, and studies in other farming systems would test whether a programme designed around the milking posture generalises to other agricultural tasks.

## 5. Conclusions

In women dairy farmers engaged in milking, both ergonomics training alone and ergonomics training combined with a 12-week structured exercise programme were associated with reduced pain and improved upper-limb function, depressive symptom scores and quality of life, and reported musculoskeletal symptom prevalence declined in both groups. The combined programme showed significantly greater between-group improvement for most of these outcomes, with the exception of the WHOQOL-BREF social relationships domain. These findings are short-term, exploratory and based entirely on self-reported outcomes obtained in a small sample from a single region under closely monitored conditions; within these limits, adding an equipment-free home exercise programme to ergonomics education resulted in greater short-term improvements in several patient-reported outcomes. Larger trials with objective adherence measures, prespecified primary analyses and longer follow-up are required before routine implementation can be recommended.

## Figures and Tables

**Table 3:** Within-group and between-group changes in continuous outcome measures.

Outcome	Group	Baseline	Week 12	Change	Within-Group *p*	Effect Size	**Baseline *p***	**Change *p***	***d***
WHOQOL-BREF general two-item facet	Control	13.12 ± 3.42	14.08 ± 2.48	0.96 ± 1.65	0.012	*r* = 1.00	0.306	<0.001	1.15
	Experimental	14.08 ± 3.13	17.20 ± 1.29	3.12 ± 2.09	<0.001	*r* = 1.00			
WHOQOL-BREF physical	Control	12.52 ± 2.03	13.50 ± 1.85	0.98 ± 0.60	<0.001	*r* = 1.00	0.005	<0.001	1.37
	Experimental	13.62 ± 1.35	15.75 ± 0.66	2.13 ± 1.02	<0.001	*r* = 1.00			
WHOQOL-BREF psychological	Control	14.45 ± 1.89	14.75 ± 1.88	0.30 ± 0.33	0.002	*r* = 1.00	0.398	<0.001	1.16
	Experimental	14.85 ± 1.38	15.78 ± 1.34	0.93 ± 0.69	<0.001	*r* = 1.00			
WHOQOL-BREF social	Control	14.45 ± 2.82	15.16 ± 2.42	0.71 ± 0.87	0.001	*r* = 1.00	0.572	0.862	0.04
	Experimental	14.77 ± 2.49	15.52 ± 2.27	0.75 ± 1.22	0.022	*r* = 0.73			
WHOQOL-BREF environment	Control	14.70 ± 2.67	15.02 ± 2.66	0.32 ± 0.45	0.005	*r* = 1.00	0.124	0.012	0.70
	Experimental	15.74 ± 1.98	16.40 ± 2.04	0.66 ± 0.51	<0.001	*r* = 1.00			
Beck Depression Inventory	Control	6.68 ± 4.73	4.92 ± 3.89	−1.76 ± 1.09	<0.001	*r* = −1.00	0.922	<0.001	−1.13
	Experimental	5.76 ± 3.24	2.24 ± 1.45	−3.52 ± 1.92	<0.001	*d_z_* = −1.84			
QuickDASH	Control	25.36 ± 17.07	12.91 ± 11.05	−12.45 ± 16.29	0.001	*d_z_* = −0.76	0.079	0.003	−0.90
	Experimental	33.73 ± 13.49	7.36 ± 11.33	−26.36 ± 14.69	<0.001	*d_z_* = −1.80			
NRS pain intensity	Control	4.16 ± 1.14	1.92 ± 0.91	−2.24 ± 0.78	<0.001	*r* = −1.00	0.039	<0.001	−2.10
	Experimental	4.80 ± 1.08	1.04 ± 0.79	−3.76 ± 0.66	<0.001	*r* = −1.00			

Values are mean ± standard deviation. WHOQOL-BREF scores are on the 4–20 metric (higher = better quality of life); higher Beck Depression Inventory, QuickDASH and NRS scores indicate worse status. Within-group tests: paired-samples *t*-test where change scores were normally distributed (Shapiro–Wilk *p* > 0.05), otherwise Wilcoxon signed-rank test; effect sizes are *d_z_* or the matched-pairs rank-biserial correlation (*r*) accordingly. Between-group tests: independent-samples *t*-test or Mann–Whitney *U* test. Cohen’s d refers to the between-group comparison of change scores. NRS, Numerical Rating Scale; QuickDASH, Quick Disabilities of the Arm, Shoulder and Hand.

**Table 4:** Results of 2 × 2 mixed-model analysis of variance (time × group).

Outcome	Time: F	Time: *p*	Time: η_p_^2^	Group: F	Group: *p*	Group: η_p_^2^	T × G: F	T × G: *p*	T × G: η_p_^2^
WHOQOL-BREF general two-item facet	58.89	<0.001	0.551	8.06	0.007	0.144	16.51	<0.001	0.256
WHOQOL-BREF physical	172.40	<0.001	0.782	15.31	<0.001	0.242	23.56	<0.001	0.329
WHOQOL-BREF psychological	65.04	<0.001	0.575	2.43	0.126	0.048	16.93	<0.001	0.261
WHOQOL-BREF social	23.53	<0.001	0.329	0.24	0.627	0.005	0.02	0.896	<0.001
WHOQOL-BREF environment	50.99	<0.001	0.515	3.33	0.074	0.065	6.14	0.017	0.113
Beck Depression Inventory	143.21	<0.001	0.749	3.39	0.072	0.066	15.91	<0.001	0.249
QuickDASH	78.34	<0.001	0.620	0.21	0.653	0.004	10.06	0.003	0.173
NRS pain intensity	859.87	<0.001	0.947	0.21	0.647	0.004	55.19	<0.001	0.535

All F tests have 1 numerator and 48 denominator degrees of freedom. η_p_^2^, partial eta squared; T × G, time × group interaction, the effect of primary interest.

**Table 5:** Analysis of covariance on week-12 scores with the corresponding baseline score as covariate.

Outcome	Adjusted Mean Difference (Exp. − Control)	95% CI	F	*p*	η_p_^2^	Homogeneity of Slopes *p*
WHOQOL-BREF general two-item facet	2.62	2.03 to 3.21	79.98	<0.001	0.630	0.001 †
WHOQOL-BREF physical	1.47	1.07 to 1.87	54.49	<0.001	0.537	<0.001 †
WHOQOL-BREF psychological	0.66	0.35 to 0.97	18.47	<0.001	0.282	0.180
WHOQOL-BREF social	0.10	−0.44 to 0.64	0.14	0.708	0.003	0.820
WHOQOL-BREF environment	0.35	0.07 to 0.64	6.22	0.016	0.117	0.835
Beck Depression Inventory	−2.05	−2.58 to −1.52	60.60	<0.001	0.563	<0.001 †
QuickDASH	−7.69	−13.94 to −1.44	6.13	0.017	0.115	0.978
NRS pain intensity	−1.25	−1.58 to −0.92	57.59	<0.001	0.551	0.970

All models have 1 and 47 degrees of freedom. A negative adjusted mean difference favours the experimental group for the Beck Depression Inventory, QuickDASH and NRS; a positive difference favours the experimental group for the WHOQOL-BREF outcomes. † The homogeneity-of-regression-slopes assumption was violated for these outcomes, so the ANCOVA estimate should be interpreted with caution and the mixed-model ANOVA and change-score analyses regarded as the primary basis for inference. η_p_^2^, partial eta squared; CI, confidence interval.

## Data Availability

The original contributions presented in this study are included in the article. Further inquiries can be directed to the corresponding author.
